# What to Do First in Accidents and Emergencies

**Published:** 1883-11

**Authors:** 


**What to do First in Accidents and Emergencies.** A manual explaining the treatment of surgical and other injuries in the absence of the physician. By CHARLES W. DULLES, M. D. Second edition, revised and enlarged, with new illustrations. Philadelphia: P. Blakiston, Son & Co., 1012 Walnut Street. 1883.

This little work furnishes brief directions for resuscitating persons in such accidents as drowning; for the treatment of foreign bodies in the eye; fits or seizures; injuries to the brain; effects of heat; effects of cold; sprains; dislocations; fractures; wounds; railroad and machinery accidents; hemorrhage; transportation of injured persons; poisons; domestic emergencies, etc. The directions are simple and judicious, and the work is just such an one as should be in every household in which there is sense enough to send for the medical man to supplement the treatment here advised.

